# Psoriasis—a Possible Candidate for Vaccination

**DOI:** 10.1080/17402520600800861

**Published:** 2006

**Authors:** Lionel Fry, Barbara S. Baker, Anne V. Powles

**Affiliations:** Faculty of Medicine Imperial College London UK


*Clinical & Developmental Immunology*, June–December 2006; 13(2–4): 361–367

Taylor & Francis

Taylor & Francis Group

### Psoriasis—a possible candidate for vaccination

LIONEL FRY, BARBARA S. BAKER, & ANNE V. POWLES

*Faculty of Medicine, Imperial College, London, UK*

### Introduction

Galen (AD133–200) was the first to use the term psoriasis, which comes from the Greek word "psora", meaning itch. Although psoriasis may cause irritation, this symptom is not one of the characteristic features of the disease.

### Epidemiology

Psoriasis is most common in the northern European countries, with an incidence of 2.9% in Denmark and 2.8% in the Faroe Isles. The average incidence in European countries is given as 2% and is slightly lower in the USA, being 1.5%. In the Middle Eastern Arab states, a similar incidence to the European countries, i.e. of 2%, has been found. In the far east, an incidence of 0.37% has been reported in China. The incidence in the Indian subcontinent has been reported as similar to Europeans in some studies, whilst in others, it is lower and similar to the Chinese. There is a higher incidence in East Africans compared to those from West Africa. Psoriasis is said to be non-existent in the native Americans and aborigines from Samoa.

### Clinical features

The two most common presentations of psoriasis are guttate (GP) and chronic plaque (CPP). The sexes are equally affected in both types of psoriasis.

GP is characterised by a sudden eruption of small red scaly papules on the trunk (GP comes from the Greek word "guttata" meaning raindrop). The limbs may also be involved. New lesions may continue to appear for the first month of the eruption, the lesions remain for a second month and during the third month, the GP lesions gradually fade. GP is most commonly seen in children, adolescents and young adults. In two-thirds of patients, there is evidence of a preceding throat infection with β haemolytic streptococci.

CPP usually presents between the ages of 15–30 years. The commonest sites are the extensor elbows, knees and scalp. The onset is insidious and the plaques are of varying size, i.e. 1–10 cm. CPP varies with the so-called activity of the disease; skin lesions may resolve spontaneously, remain the same for years, or gradually increase in size with new ones appearing, which may eventually coalesce to involve all the skin. In patients with stable CPP, acute GP flares may occur, i.e. small papules appear on the trunk and limbs. In this situation, the GP lesions may resolve after three months, but the plaques remain as before the GP flare.

### Natural history

GP usually has a good prognosis and resolves after three months. However in a small number of patients, the GP lesions do not resolve but progress to CPP. In addition, even in those patients with GP, there is a risk that these patients will develop CPP in later life. CPP, however, has a variable prognosis. In mild disease, in approximately 50% of patients, a spontaneous or therapy-induced remission will occur. However, recurrence of the disease is high. Permanent remission occurred in only 17% of patients over a 20 year follow-up.

### Histology

Psoriasis is characterised by thickening of the epidermis, due to hyperplasia of the keratinocytes.

Correspondence: L. Fry, Faculty of Medicine, Imperial College, 96 Harley Street, London W1G 7HY, UK. Tel: 44 207 935 2421. Fax: 44 207 224 0656. E-mail: l.fry@imperial.ac.uk

ISSN 1740-2522 print/ISSN 1740-2530 online © 2006 Taylor & Francis
DOI: 10.1080/17402520600800861

There is also failure of keratinocyte maturation with loss of the granular layer and an abnormal stratum corneum consisting of loosely packed keratin and infiltration with neutrophils. There is elongation of the basement membrane with down growth of the rete ridges to accommodate the hyperplasia of the epidermal cells. In the dermis, there is an increase in size and tortuosity of the capillaries in the dermal papillae and a lymphocytic infiltrate. Neutrophil microabscesses may be seen in the upper epidermis.

### Genetics

### *Family studies*

It has been observed for many years that psoriasis tends to run in families. Studies have suggested that psoriasis is inherited as a Mendelian dominant with incomplete penetrance of the gene(s). However, others have interpreted the family studies over generations to imply a recessive mode of inheritance with 90% penetrance. More recent studies have now suggested that psoriasis is a polygenic disorder. In addition, environmental factors are also important in the expression of the psoriasis and it is now considered a complex multifactorial disease.

### *Census studies*

There have been two large census studies in psoriasis, one in the Faroe Isles in which 30,000 individuals were examined (Lomholt 1963) and the other in central Sweden in which 40,000 individuals were seen (Hellgren 1967). In the latter, it was found that 6.4% of relatives of patients with psoriasis were affected compared to 1.96% of the general population. In the study of the closed community of the Faroe Isles, 91% of patients with psoriasis had a family history. Recent analysis of the data from these two studies supports the current concept that psoriasis is a multifactorial disorder and that monogenic types of inheritance are now excluded.

### *Twin studies*

These have strongly supported the role of inheritance in psoriasis as the concordance for monozygotic twins is 70% and dizygotic twins 14%. Twin studies have also shown that the clinical type of psoriasis, age of onset, course and severity are to a large extent determined by genetic factors.

### *HLA*

Class I HLA studies have shown association with B13, B17, B37 and B57. However, the strongest association has been shown with Cw6. The association with the B antigens is considered to be due to linkage disequilibrium with Cw6.

The highest incidence of Cw6 in psoriasis is in Caucasians and the reported incidence varies from 36 to 84%, the average being 60 to 65%. The incidence in Asians is considerably lower, having been reported as 26% in a study in Japanese and 17% in Chinese. However, the incidence of Cw6 is still raised in Asians, as the incidence in a control population in Asians is 1–2% compared to 10–15% in Caucasians.

Clinical features of psoriasis show a relationship to Cw6 in Caucasians. Cw6 is associated with early onset, GP eruptions, increased severity and a positive family history (Hensler and Christophers 1985; Gudjonsson et al 2002). However, the association with GP psoriasis is not absolute and this pattern of the disease can occur in patients who are not Cw6-positive (Fry et al. 2006).

Class II antigens, DR4 and DR7 have also shown an association with psoriasis. The incidence for DR7 is the highest and is approximately 60% in Caucasians (control population, 9–10%). Recently, a negative association was reported for DR15 (Baker et al 2003). Patients expressing this HLA antigen had mild disease, late onset and no GP eruptions.

### *Chromosome loci and genes*

There are now seven loci reported for psoriasis, PSORS1 on 6p21.3, PSORS2-17q25, PSORS3-4q.34, PSORS4-1q21, PSORS5-3q, PSORS6-19p and PSORS7-1p. These findings were described in different ethnic groups and not all have been confirmed. The locus that has been found in most studies is that on 6p21.3 and this region includes HLA Cw6. However, as yet, no gene mutation has been definitely identified on this locus using microsatellite markers and single nucleotide polymorphisms (SNPs). Some studies have implied that the polymorphism is within Cw6 itself, but most have reported the region associated with psoriasis to be telomeric to Cw6 and a few to be centromeric. Although sequencing of Cw6 has shown an association between alanine at position 73 and psoriasis in Japanese patients, this was not been confirmed in patients of European extraction. Recently, gene mutations have been reported in regions containing SLC9ARI on the 17q25 locus (Helms et al. 2003) and SLC12A8 on the 3q locus (Hewett et al. 2002). SLC9ARI is concerned with transport of ions across cell membranes and immune synapse formation in T cells and is adjacent to a putative binding site for the transcription factor RUNXI. It has also been proposed that Jun proteins, the genes for which are located at the 19p locus, may be implicated in psoriasis (Zenz et al. 2005). Abrogation of Jun proteins has been shown to induce the production of cytokines and chemokines, which results in proliferation of keratinocytes and infiltration of the epidermis with inflammatory cells.

### Aetiology

Factors that may induce and/or aggravate psoriasis include streptococcal infections, stress, trauma to the skin (Koebner phenomenon), drugs, (particularly lithium), alcohol, smoking, obesity and climate. Stress and streptococcal infections have the most clear-cut relationship to inducing or aggravating existing psoriasis. In this article, the role of streptococcal infections will be discussed in detail.

### Streptococcal infections

It was noted over 50 years ago that an acute sore throat often preceded the appearance of psoriasis (Norholm-Pederson 1952). It was subsequently shown that in patients with psoriasis, there was a significantly higher incidence of a positive streptococcal agglutination test compared to patients with other skin diseases. The association of streptococcal throat infections with GP is much stronger than that with CPP. Either one or more of the following is present in over half of patients with GP: history of a sore throat approximately two weeks prior to the eruption, a positive throat swab for β haemolytic streptococci and a raised anti-streptolysin titre. Confirmation of a streptococcal infection in the throat in GP is difficult because the patients present a few weeks after the infection when the organisms are no longer present on the surface of the tonsil. This may be partly due to the ability of streptococci to penetrate tonsillar epithelial cells where they may persist indefinitely, out of reach of the commonly used antibiotic, penicillin. In addition, the anti-streptolysin titre takes 10–14 days to appear in the blood and will begin to fall after six weeks contributing to the difficulty of confirming that a streptococcal infection preceded the initiation of skin lesions.

It has been shown that not only group A, β haemolytic streptococci can trigger psoriasis but also groups C and G (Belew et al. 1985). No particular streptococcal serotype is linked to the induction of psoriasis (Telfer et al. 1992).

There is evidence that CPP is also related to streptococcal throat infections. First, it has been reported that in patients with CPP recurrent sore, throats are three times more common than in sex and age matched controls (Wardrop et al. 1998). Second, in CPP β haemolytic streptococcal organisms (groups A, C or G) are present in 20–30% of subjects (Cohen-Tervaert and Esseveld 1970). Third, 25% of CPP patients have raised anti-streptolysin titres (Cohen-Tervaert and Esseveld 1970). The fact that evidence of streptococcal organisms in the throat can be found for only 25% of patients may suggest that streptococci are more commonly located intracellularly in tonsils or associated lymphoid tissue, where they could act as a source of antigen to maintain the psoriatic process.

### Immunology

### *Guttate psoriasis*

The initiation of GP has been shown to be associated with an influx of activated $CD4^+$T lymphocytes into the dermis and epidermis, whilst resolution is associated with an influx of activated $CD8^+$T cells into the epidermis. In the initiation phase, activated $CD4^+$T cells are in close apposition to the dendritic processes of APCs, whilst in resolution, they are replaced by activated $CD8^+$T cells. It is likely that the hyperproliferation of the keratinocytes is induced by cytokines secreted by activated $CD4^+$T cells; in contrast, $CD8^+$T cells acting as regulatory cells probably mediate resolution. In GP, it has been shown that there is increased usage of TCR Vβ2 and Vβ5.1 by infiltrating T cells suggestive of a super-antigen effect (Lewis et al. 1993).

### *Chronic plaque psoriasis*

In CPP, there is an influx of activated $CD4^+$T cells in the dermis and of both activated $CD4^+$ and $CD8^+$ T cells in the epidermis. It had been assumed that the $CD4^+$T cells were effector cells and that the $CD8^+$ T cells acted as suppressor or regulatory cells. However, recently it has been suggested that the $CD8^+$T cells may have an effector role in CPP (Gudjonsson et al. 2004). In CPP, oligoclonality of infiltrating T cells has been demonstrated suggesting that that they are activated by a specific antigen.

As a result of the initial studies of T cells in psoriasis, a hypothesis was put forward that "psoriasis is a hyperproliferative disorder of keratinocytes mediated by T lymphocytes" (Valdimarsson et al. 1986).

### Nature of the antigen

Thus, it is accepted that the hyperproliferation of the keratinocytes is due to cytokines produced by activated T cells. What has still to be determined is the nature of the antigen that activates the T cells.

In GP, the increased usage of particular Vβ families suggests a superantigen effect. Streptococcal organisms produce a number of superantigens including exotoxins A, C, F, X and SMP2. Analysis of the TCR in GP psoriasis has revealed extensive region diversity supporting the activation of T cells by superantigens (Leung et al. 1995).

In CPP, there is oligoclonality of the TCR indicative of a specific antigen. The candidates for a streptococcal antigen include cellular proteins, such as T and M proteins, cell wall and cell membrane proteins.

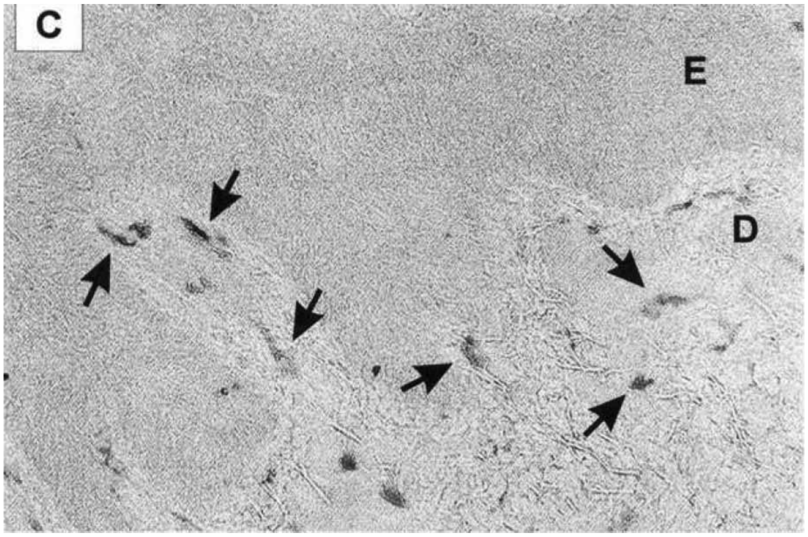

Figure 1. Macrophages containing PG in lesional psoriasitic skin. Increased number of 2E9-positive cells are present in the upper dermis and dermal papillae. E = epidermis; D = dermis; and 2E9 is a monoclonal antibody anti-PG $IgG_3$ kindly supplied by Dr Jon Laman of Erasmus Medical Centre, Rotterdam.

It has been shown that T cell lines cultured from psoriasis lesions respond to whole streptococcal, cell wall and cell membrane extracts (Baker et al. 2001; Brown et al. 2001). The response is measured by proliferation and/or IFN-$\gamma$ production. Peptidoglycan is the major constituent of the cell wall; our group therefore tested skin T cell responses to streptococcal peptidoglycan (PG) and found specific HLA-DR restricted responses to this antigen (Baker et al. 2006). We have also recently shown that cells containing peptidoglycan are significantly increased in the dermis of psoriatic lesions (Figures 1 and 2), and that a proportion of these cells are macrophages (Baker et al. 2006). It is possible that if streptococcal organisms are present and persist in the lymphoid tissue in the throat, that peptidoglycan is released and taken up by macrophages, which carry the bacterial antigen to tissues including the skin (Figure 3). In addition, T cells present in the tonsils would be primed to streptococcal antigens including peptidoglycan and induced to express the skin homing receptor, cutaneous lymphocyte antigen (CLA), by interaction with streptococcal toxins. These primed tonsillar T cells would then be able to infiltrate the skin where they could again encounter peptidoglycan. Activation of T cells by peptidoglycan would result in the production of cytokines, which in turn would induce keratinocyte hyperproliferation.

Another theory proposed for psoriasis is that streptococcal M protein induces the disease. It has been suggested that M protein initiates the psoriatic process and because of homology between M protein

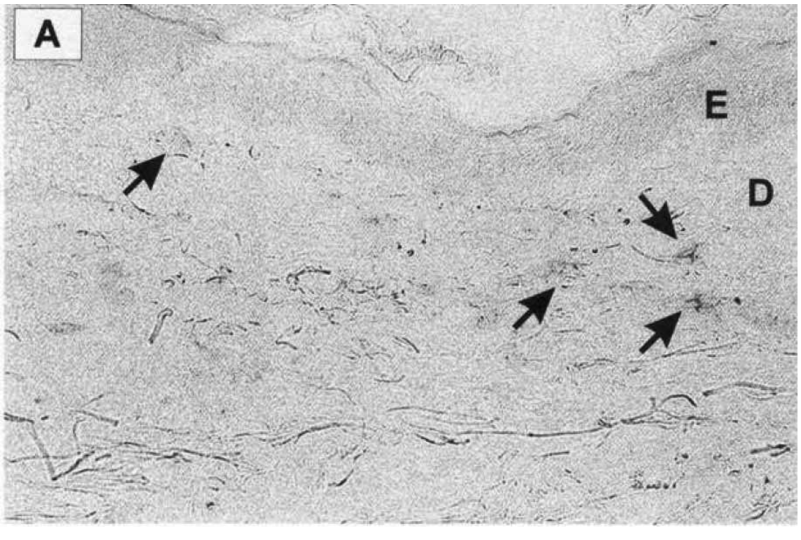

Figure 2. In comparison, normal skin showing very few positive 2E9 cells. E = epidermis; and D = Dermis

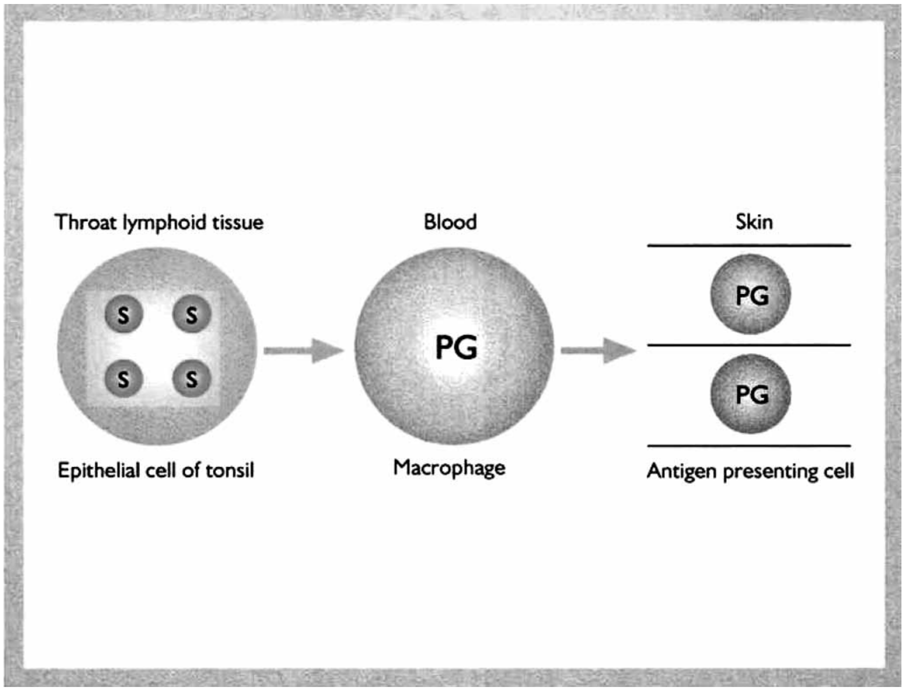

Figure 3. Possible pathway of streptococcal PG from the throat to the skin.

and keratin 17, the disease is maintained by the epidermal antigen, which is up-regulated in the skin in psoriasis (Sigmundsdottir et al. 1997; Gudmundsdottir et al. 1999). In support of this autoimmune cross-reactive hypothesis, circulating CLA$^+$ CD8$^+$T cells in Cw6-positive psoriasis patients were shown to respond to a predicted Cw6-binding peptide from keratin 17 that shares the ALEEAN sequence with M protein (Johnston et al. 2004). In contrast, a response to M protein has not been detected for CD4$^+$T cell lines cultured from psoriasis skin lesions (Baker et al. 2001; Brown et al. 2001).

### Psoriasis and Crohn's disease

Patients with Crohn's disease (CD) and their first-degree relatives, have a fivefold increase in psoriasis (Yates et al. 1982; Lee et al. 1990). Thus, there appears to be a genetic link between the two diseases. Interestingly both psoriasis and CD have susceptibility loci on chromosomes 3, 4, 5 and 16 (Najarian and Gottlieb 2003). In CD, a mutation has been found on chromosome 16q21 in the gene for NOD2 (CARD15). NOD2 is an innate immune receptor for muramyl dipeptide (MDP) a constituent of peptidoglycan. It has been found in CD that in patients with NOD2 mutations, there is an altered immunological response to MDP; peripheral blood mononuclear cells show decreased production of the pro-inflammatory cytokines, TNF-$\alpha$ and 1L-1$\beta$. As a consequence, there is a defective recruitment of neutrophils, which leads to a decreased immune response to bacteria (Van Heel et al. 2005). However in another recent study, it has been shown, there is a weaker immune response to bacterial extracts, compared to controls, which was independent of NOD2 mutations (Marks et al. 2006). It has been proposed that the defects in the innate immune system in CD result in failure of early immune pathogen clearance and explains the abnormal adaptive immune response to microbial antigens.

Although the NOD2 mutations have not been found in psoriasis (Borgiani et al 2002; Young et al. 2003), mutations in other receptors in the innate immune system may exist. Recently, four peptidoglycan recognition proteins (Pglyrp) have been described, the genes for three of which are at loci for psoriasis; Pglyrp-2 is found on 19p13.12 (PSORS6) and Pglyrp-3 and Pglyrp-4 on 1q21 (PSORS4). A recent study has shown a possible association of Pglyrp-3 and Pglyrp-4 with psoriasis in the transmission disequilibrium test (TDT); however, this was not confirmed in the case-control test (Sun et al. 2006).

As yet, there is no evidence of impaired innate immunity in psoriasis but only limited investigations have been carried out. Because of the similarity between CD and psoriasis, i.e. they are both chronic inflammatory diseases and there is accumulating evidence that both are induced by bacterial components, it is possible that there is a defect in the innate immune system in psoriasis. Furthermore, bacterial peptidoglycan also appears to be a candidate in psoriasis following the demonstration of an adaptive T cell immune response to streptococcal PG in skin lesions (Baker et al. 2006).

### Conclusions

There is now substantial evidence, both clinical and laboratory based, that streptococci are involved in the pathogenesis of psoriasis. Whether the primary site of pathogenesis is in the lymphoid tissue in the throat, or in the skin, has yet to be determined.

Streptococcal PG is a good candidate for an aetiological factor in psoriasis as it has the ability to affect both innate and adaptive immune responses. As mentioned above, evidence already exists for an adaptive immune response (Baker et al. 2006). If it is accepted that streptococci are involved in the pathogenesis of psoriasis, then vaccination may well have a role to play in prevention and management of the disease. However, it should be stressed that streptococci may not be the only microorganisms that induce or maintain the disease. Other possible candidates include *Staphylococcus aureus*, *Candida albicans* (Leung et al. 1993) and *Pityrosporum orbiculare* (Baker et al. 1997). The type of vaccines to be developed for combating *Streptococcus pyogenes* in psoriasis will have to be left to vaccinologists. However, it is pertinent that a vaccine against *S. pneumoniae*, based on the cell wall (PG), already exists and has shown to be effective (Prymula et al. 2006).

Psoriasis is considered to be a complex multi-factorial disease and genetic defects involving clearance of microorganisms and/or hyperproliferation of keratinocytes may be involved. It is salutary to recall that over 100 years ago, Radcliffe Cocker in his Textbook of Dermatology stated that psoriasis was due to bacteria on the skin. This may still prove to be correct. Current treatments are focused on suppressing the immune system; elimination of, or protection from, bacteria by vaccination may represent a new and possibly more effective treatment for this common skin disease.

### Acknowledgements

We gratefully acknowledge Dr Jon Laman, Department of Immunology, Erasmus Medical Centre, Rotterdam, The Netherlands, for supplying the monoclonal antibody 2E9 raised against peptidoglycan.

### References

Baker BS, Powles AV, Garioch JJ, Hardman C, Fry L. 1997. Differential T cell reactivity to round and oval forms of pityrosporum in the skin of patients with psoriasis. Br J Dermatol 136:319–325.

Baker BS, Brown DW, Fischetti VA, Ovigne JM, Porter W, Powles AV, Fry L. 2001. Skin T cell proliferative responses to M protein and other cell wall and membrane proteins of group A streptococci in chronic plaque psoriasis. Clin Exp Immunol 124:516–521.

Baker BS, Ovigne JM, Fischetti VA, Powels AV, Fry L. 2003. Reduced gamma IFN responses associated with HLA-DR15 presentation of streptococcal cell wall proteins to dermal Thl cells in psoriasis. J Clin Immunol 23:407–415.

Baker BS, Laman JD, Powles AV, van der Fits L, Voerman JSA, Melief M-J, Fry L. 2006. Peptidogclycan and peptidoglycan-specific Thl cells in psoriasis. J Pathol, In press.

Belew PW, Wannamaker LW, Johnson D, Rosenberg EW. 1985. Beta haemolytic streptococcal types associated with psoriasis. In: Kimera K, Kotanie S, Shiokawa Y, editors. Recent advances of streptococcal diseases. UK: Reedbooks Ltd. p 334.

Borgiani P, Vallo L, D'Apice MR, Giardina E, Pucci S, Capon F, Nis S, Chimenti S, Pallone F, Novelli G. 2002. Exclusion of CARD15/NOD2 as a candidate susceptibility gene to psoriasis in the Italian population. Eur J Dermatol 12:540–542.

Brown DW, Baker BS, Ovigne JM, Fischetti VA, Hardman C, Powles AV, Fry L. 2001. Non-M protein(s) on the cell wall and membrane of group A streptococcus induce(s) IFN gamma production by dermal CD4 + T cells in psoriasis. Arch Dermatol Res 293:165–170.

Cohen-Tervaert WC, Esseveld HA. 1970. A study of the incidence of haemolytic streptococci in the throat in patients with psoriasis with reference to their role in the pathogenesis of the disease. Dermatologica 140:282–290.

Fry L, Powles AV, Corcoran S, Rogers S, Ward J, Unsworth DJ. 2006. HLA Cw*06 is not essential for streptococcal induced psoriasis. Br J Dermatol, In Press.

Gudjonsson J, Karason A, Antonsdottir A, Runarsdottir EH, Gulcher JR, Stefansson K, Valdimarsson H. 2002. HLA-Cw6 positive and HLA-Cw6 negative patients with psoriasis vulgaris have distinct clinical features. J Invest Dermatol 118:362–365.

Gudjonsson J, Johnston A, Sigmundsdottir H, Valdimarsson H. 2004. Immunopathogenic mechanisms in psoriasis. Clin Exp Immunol 35:1–8.

Gudmundsdottir AS, Sigmundsdottir H, Sigurgeirsson B, Good MF, Valdimarsson H, Jonsdottir I. 1999. Is an epitope on keratin 17 a major target for autoreactive T lymphocytes in psoriasis. Clin Exp Immunol 117:580–586.

Hellgren L. 1967. Psoriasis. Stockholm: Almqvist & Wiksell.

Helms C, Cao L, Krueger JG, Wijsman EM, Chamion F, Gordon D, Hefferman M, Daw JA, Robarge J, Ott J, Kwok PY, Menter A, Bowcock AM. 2003. A putative RUNX1 binding site variant between SLC9A3R1 and NAT9 is associated with susceptibility in psoriasis. Nat Genet 35:349–356.

Hensler T, Christophers E. 1985. Psoriasis of early and late onset: Characteristics of two types of psoriasis. J Am Acad Dermatol 13:450–456.

Hewett D, Samuelsson L, Polding I, Enlund F, Smart D, Cantone K, See CG, Chadha S, Inerot A, Enerback C, Montgomery D, Christodolou C, Robinson P, Matthews P, Plumpton M, Wahlstrom J, Swanbeck G, Martinsson T, Roses A, Riley J, Purvis I. 2002. Identification of a psoriasis susceptibility candidate gene by linkage disequilibrium mapping with a localised single nucleotide polymorphism map. Genomics 79:305–314.

Johnston A, Gudjonsson JE, Sigmundsdottir H, Love TJ, Valdimarsson H. 2004. Peripheral blood T cell responses to keratin peptides that share sequences with streptococcal M proteins are largely restricted to skin-homing $CD8^{+}$T cells. Clin Exp Immunol 138:83–93.

Lee FI, Bellamy SV, Francis C. 1990. Increased occurrence of psoriasis in patients with Crohn's disease and their relatives. Am J Gastroenterol 85:962–963.

Leung DYM, Walsh P, Giorno R, Norris DA. 1993. A potential role for superantigens in the pathogenesis of psoriasis. J Invest Dermatol 100:225–228.

Leung DYM, Travers JB, Giorno R, Norris DA, Skinner R, Aelion J, Kazemi LV, Kim MH, Trumble AE, Kobt M. 1995. Evidence of streptococcal superantigen-driven process in acute guttate psoriasis. J Clin Invest 96:2106–2117.

Lewis HM, Baker BS, Bokt S, Powles AV, Garioch JJ, Valdimarsson H, Fry L. 1993. Restricted T-cell receptor V beta gene usage in the skin of patients with guttate and chronic plaque psoriasis. Br J Dermatol 129:514–520.

Lomholt G. 1963. Psoriasis: Prevalence, spontaneous course and genetics. Copenhagen: G.E.C. Gad.

Marks DJB, Harbord MWN, Macallister R, Rahman FZ, Young J, Al-Lazikani B, Lees W, Novelli M, Bloom S, Segal AW. 2006. Defective acute inflammation in Crohn's disease: A clinical investigation. Lancet 367:668–678.

Najarian DJ, Gottlieb AB. 2003. Connections between psoriasis and Crohn's disease. J Am Acad Dermatol 48:805–821.

Norholm-Pederson A. 1952. Infections and psoriasis. Acta Dermatol Venerol 32:159–167.

Prymula R, Peters P, Chrobok V, Kriz P, Novakova E, Kaliskova E, Kohh I, Lommel P, Poolman JH, Prieels J-P, Schuerman L. 2006. Pneumococcal capsular polysaccharides conjugated to protein D for prevention of acute otitis media caused by both streptococcus pneumoniae and non typable haemophilius influenzal: A randomised double-blind efficacy study. Lancet 367:740–748.

Sigmundsdsottir H, Sigurgeirsson B, Troye-Blomberg M, Good MF, Valdimarsson H, Jonsdottir I. 1997. Circulating T cells of patients with active psoriasis respond to streptococcal M peptides sharing sequences with human epidermal keratins. Scand J Immunol 45:688–697.

Sun C, Mathur P, Dupuis J, Tizard R, Ticho B, Crowell T, Gardner H, Bowcock AM, Carulli J. 2006. Peptidoglycan recognition proteins Pglyrp-3 and Pglyrp-4 are encoded from the epidermal differntiation complex and are candidates for PSORS4 locus on chromosome 1q21.

Telfer NR, Chalmers RJ, Whale K, Coleman G. 1992. The role of streptococcal infection in the initiation of guttate psoriasis. Arch Dermatol 128:39–52.

Valdimarsson H, Baker BS, Jonsdottir I, Fry L. 1986. Psoriasis, a disease of abnormal keratinocyte proliferation mediated by T cells. Immunol Today 7:256–257.

Van Heel DA, Ghosh S, Butter M, Hunt KA, Lundberg AMC, Ahmad T, McGovern DPB, Onnie C, Negoro K, Goldthorpe S, Foxwell BMJ, Matthew CG, Forbes A, Jewell DP, Playford RJ. 2005. Muramyl dipeptide and toll-like receptor sensitivity in NOD2 associated Crohn's disease. Lancet 365:1794–1796.

Wardrop P, Weller R, Marais J, Kavanagh G. 1998. Tonsillitis and chronic psoriasis. Clin Otolaryngol 23:67–68.

Yates BM, Watkinson G, Kehman A. 1982. Further evidence for an association between psoriasis and Crohn's disease. Br J Dermatol 106:323–330.

Young C, Allen MH, Cuthbert A, Ameen M, Veal C, Leman J, Bad AD, Kirby B, Griffiths CEM, Trembath RC, Matthew CG, Barker JN. 2003. A Crohn's disease-insertion polymorphism (3020 ins C) in NOD2 gene is not associated with psoriasis vulgaris, palmo-plantor pustular psoriasis or guttate psoriasis. Exp Dermatol 12:506–509.

Zenz R, Efert R, Kerner L, Florin L, Hummerich L, Mehic D, Scheuch H, Angel P, Tschachler E, Wagner EF. 2005. Psoriasis-like skin disease and arthritis caused by inducible epidermal deletion of Jun proteins. Nature 437:369–375.

